# Smoking cessation rates in elderly and nonelderly smokers after participating in an intensive care smoking cessation camp

**DOI:** 10.1097/MD.0000000000029886

**Published:** 2022-07-29

**Authors:** Jae-Kyeong Lee, Yu-Il Kim, Sun-Seog Kweon, In-Jae Oh, Yong-Soo Kwon, Hong-Joon Shin, Yu-Ri Choe, Ha-Young Park, Young-Ok Na, Hwa-Kyung Park

**Affiliations:** a Department of Internal Medicine, Chonnam National University Medical School and Hospital, Gwangju, South Korea; b Department of Preventive Medicine, Chonnam National University Medical School and Hwasun Hospital, Hwasun, South Korea; c Department of Internal Medicine, Chonnam National University Medical School and Hwasun Hospital, Hwasun, South Korea; d Department of Family Medicine, Chonnam National University Medical School and Hwasun Hospital, Hwasun, South Korea.

**Keywords:** abstinence, elderly, intensive care smoking cessation camp

## Abstract

Since it is a widely known fact that smoking cessation is beneficial physically and cognitively, efforts should be made to enable smokers to quit smoking through policy. Intensive care smoking cessation camps generally show a high smoking cessation success rate, but research is needed to determine which smokers should be admitted due to costeffectiveness. Although many studies have been conducted to find factors related to smoking cessation success, there is still controversy about the will and success rate of smoking cessation of elderly smokers. We performed this study to determine behavior characteristics and smoking cessation success rates in nonelderly and elderly smokers who participated in an intensive care smoking cessation camp.

Heavy smokers participating in an intensive care smoking cessation camp at Chonnam National University Hospital between the August 2015 and December 2017 were classified into elderly (age ≥65 years old) or nonelderly (age <65 years old) groups after excluding missing data. Smokers were followed up at 4 weeks, 6 weeks, 12 weeks, and 6 months from the start of abstinence by self-report, measurement of carbon monoxide expiration levels or cotinine testing.

A total of 351 smokers were enrolled in the study. At the 6-month follow-up, 56 of 107 (52.3%) elderly smokers and 109 of 244 (44.7%) nonelderly smokers continued to abstain from smoking. Elderly smokers showed a higher smoking cessation rate than that of nonelderly smokers, but it was not statistically significant (OR = 1.36, 95%CI: 0.862, 2.145). The most common causes of cessation failure in both groups were stress and temptation, followed by withdrawal symptoms.

Smoking cessation rates in the elderly are comparable to that in the nonelderly after an intensive care smoking cessation camp. Intensive care smoking cessation camps can help both elderly and nonelderly smokers who intend to quit smoking by providing motivation, education and medication. Smoking cessation should be strongly recommended regardless of age.

## 1. Introduction

Cigarette smoking is the leading cause of death from cancer, chronic obstructive pulmonary disease, and heart disease. Absolute risk differences for these diseases between never smokers and current smokers are higher in the elderly.^[1]^ In 2015, approximately 6 million deaths worldwide were attributed to smoking, with this figure expected to rise annually reaching approximately 8 million deaths by 2030.^[2]^

Smoking can also cause problems in areas such as cognitive function and quality of life as well as physical problems. Balfour et al reported in 2006 that patients with hepatitis C virus (HCV) who smoke had a lower score of physical and mental subscale, which means quality of life, than patients with HCV who do not smoke, and argued that smoking behavior should be evaluated and smoking cessation should be recommended for the care of patients with HCV.^[3]^ Almeida et al. reported that people who failed to quit smoking had lower cognitive function scores and a greater loss of gray matter in the brain than that of those who succeeded in quitting smoking through a 2-year observational study in 2010.^[4]^ Furthermore, a study by Fernandes published in 2017 announced that the spatial vision of smokers was less sensitive than that of nonsmokers, and it is thought that the components of tobacco generate free radicals and cause macular degeneration, which affect visual spatial processing.^[5]^

Benefits of abstinence, including preventing severe illness and maintaining good health, are apparent at any age.^[6]^ As an aging society progresses in many countries, the impact of smoking cessation in the elderly is becoming increasingly important.^[7]^ Clinicians are required to recommend that patients quit smoking.^[8]^ However, quitting smoking has been recognized as a very difficult task, and nicotine contained in cigarettes has been thought to play an important role because of its action in stimulating mesolimbic dopamine neurons and causing nicotine dependence.

In addition to dopamine, serotonin and glutamate were also found to play important roles in tobacco addiction. Balfour concluded in 2006 that smoking was reinforced by conditioned stimuli at the time of smoking, not simply due to nicotine dependence and dopamine reward.^[9]^ In a study conducted by Benowitz et al in 2012, when nicotine contained in cigarettes was gradually reduced for 6 months, the smoker plasma cotinine level was also less detected, indicating that the reduced nicotine in cigarettes did not cause compensation, which supported Balfour conclusion for the relationship between neurotransmitters and tobacco addiction. The United States Food and Drug Administration regulates the amount of nicotine contained in cigarettes by policy, and efforts are needed to match the actual policy with the results of academic research.^[10]^

In Korea, the government-led smoking cessation service started systemically in 2005 when smoking cessation clinics were opened at public health centers across the country. In addition, 18 regional smoking cessation support centers have been installed, 1 for each region, providing residential smoking cessation camp programs and smoking cessation treatment services, thereby increasing the professionalism and accessibility of services at the national level since 2015.^[11]^ In a cohort analysis of 4327 smokers conducted at Mayo Clinic from 2004 to 2007, smokers who received residential treatment showed significantly higher smoking cessation success rates than that of smokers who received outpatient treatment, even though their dependence on nicotine was more severe.^[12]^ Nevertheless, despite the high smoking cessation success rates of smoking cessation camps for heavy smokers, there are critical views in terms of cost effectiveness and scope of application.

It is important to study which factors make patients more successful in quitting smoking in order to develop and verify the effectiveness of an intensive program; such a program can expand the target population while reducing future costs. Many studies have been conducted worldwide to identify the factors affecting smoking cessation. Nonetheless, it has remained inconclusive whether age affects the success of smoking cessation. According to a study based on a telephone survey of 9,000 adult smokers in the United Kingdom, the United States, Canada, and Australia in 2005, older smokers are insensitive to the harm of quitting smoking, lack confidence, and do not want to quit.^[13]^ However, a cross-sectional study of 7839 adults aged 19-65 years from 2007 to 2012 conducted in Republic of Korea reported that older age is associated with successful smoking cessation.^[14]^

The success rate of smoking cessation among older people is controversial. Since the physical and mental benefits of quitting smoking are clear for the elderly, if the success rates of smoking cessation in intensive care smoking cessation camps are high, policies such as actively recommending smoking cessation camps for elderly heavy smokers can be considered. Thus, the aim of this study is to determine behavior characteristics and smoking cessation success rates in nonelderly and elderly smokers after participating in an intensive care smoking cessation camp.

## 2. Materials and Methods

### 2.1. Study participants and data collection

**Figure 1.**
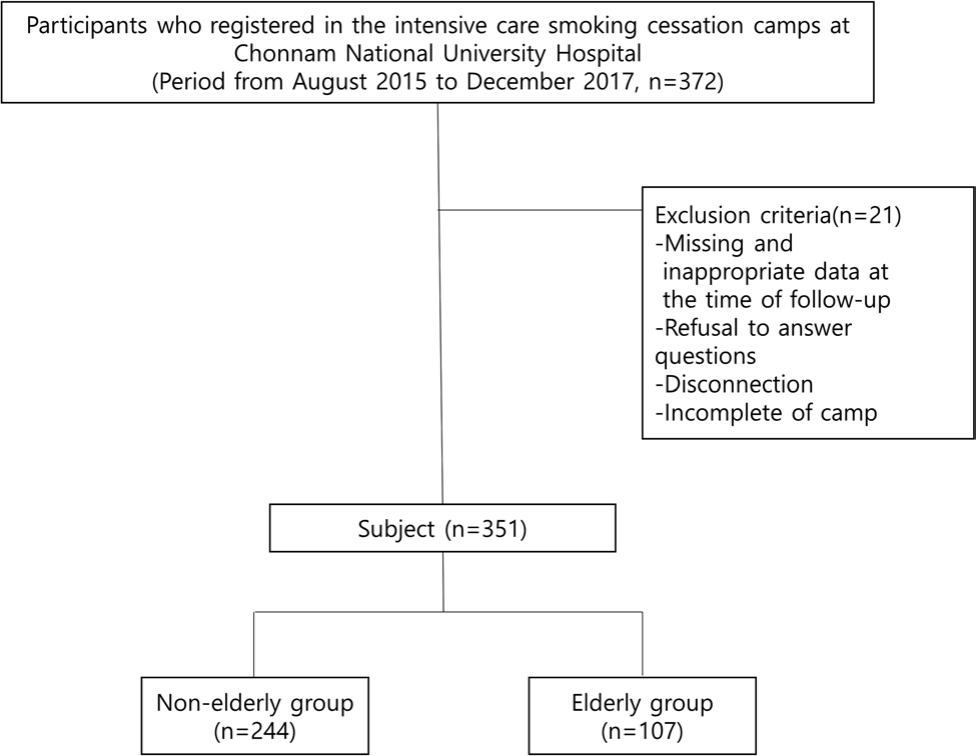
A total of 372 smokers were registered in the intensive care smoking cessation camp at Chonnam National University Hospital from August 2015 to December 2017, and 21 smokers were excluded due to missing and inappropriate data. The remaining 351 patients were classified as elderly and nonelderly based on the age of 65 years, and the success rates of smoking cessation were compared.

Data were obtained from the National Tobacco Control Center from August 2015 to December 2017 for heavy smokers registered in the intensive care smoking cessation camp at Chonnam National University Hospital. The participants of the camp were smokers who had a history of smoking-related diseases (including malignant tumors, chronic lung disease, cardiovascular disease), smoked cigarettes for >20 years, and had failed to quit smoking twice or more. Of the 372 individuals who had registered for the program, 20 were excluded due to either refusal to answer questions, disease and death, or disconnection, and 1 was because she failed to complete smoking cessation camp. The remaining 351 patients were included in the study for analysis. To compare the smoking cessation success rate according to age, smokers were classified into an elderly group and a nonelderly group based on the age of 65 years (Fig. 1). The study protocol was approved by the Institutional Review Board of Chonnam National University Hospital (No. CNUH-2021-414), and the requirement for informed consent was waived because of the retrospective nature of this study. This study complied with the Declaration of Helsinki.

### 2.2. Overview of the intensive care smoking cessation camp program

Intensive care smoking cessation camps are a program conducted at a local smoking cessation support center for 4 nights and 5 days for heavy smokers who have the will to quit but are finding it difficult. Programs of intensive care smoking cessation camps include dealing with the harmful effects of cigarettes, strengthening the motivation to quit smoking, understanding antismoking drugs, training in stress management and anger management, and understanding the marketing strategies of tobacco companies. Each individual can participate in the smoking cessation camp by visiting the local smoking cessation support center or using the online application. Participants are required to pay an entry fee of about 1000 dollars, but are eligible for a full refund upon completion of the camp. Youths <19 years, the elderly >65 years, the disabled, recipients of medical benefits (such as those receiving basic livelihood benefits), the second-highest class (those whose recognized income is ≤50% of the median income), and soldiers and police officers performing military service are exempt from cost. A souvenir is provided for completion and success. Services for smoking cessation maintenance and practice are provided for 6 months through onsite or phone counseling. Details of the program are shown in Table 1.^[11]^

**Table 1:** Special treatment type at intensive care smoking cessation camp: program contents.

Task	Timepoint	Contents
Application to smoking cessation camp		① Referral letter form hospital or clinic ② Submission of application form to smoking cessation camp (on site visit, online)
Prescreening	1_Week before camp	① Evaluation of application form to smoking cessation camp (personal information, smoking history, motivation, etc) ② Prescreening (telephone): check potential problems in camp participation and motivation ③ Check application fee (certification documents for fee waivers) ④ Selection of participant: information provision for precamp prescription of smoking cessation medication; reservation for smoking cessation clinic and consultation
Registration for camp	Day 1	① Rules for smoking cessation camp participation, informed consent for participation, and informed consent for personal information ② Registration card: smoking status evaluation (amount, duration, etc), smoking habit, self-efficacy, contraindications for nicotine replacement therapy, nicotine dependence test, etc
Participation in camp	Day 1	① Smoking cessation consultation: prescription based on smoking status evaluation (amount, duration, etc) ② Smoking cessation education: education on smoking cessation medication ③ Psychologic counseling: motivation for camp participation
	Day 2	① Rounding: check smoking status (carbon monoxide monitoring), craving for smoking, effect and side effect of smoking cessation medication, etc ② Health status check and basic fitness test: blood pressure, body fat, hemoglobin, liver function, renal function, cholesterol, lung function, basic fitness level, nutrition status, etc ③ Smoking cessation education: theme-healthy lung, diet plan after smoking cessation ④ Psychologic counseling: consolidation of motivation for smoking cessation (confirmation of reasons of smoking cessation)
	Day 3	① Rounding: check smoking status (carbon monoxide monitoring), craving for smoking, effect and side effect of smoking cessation medication, etc ② Exercise program: aerobic, stretching, muscle power ③ Smoking cessation education: theme-harms of smoking, smoking cessation policy, etc ④ Psychologic counseling: dealing with ambivalence in smoking cessation
	Day 4	① Counseling about health check-up results: explanation of the results of health check-up Identify the health status related to smoking, motivation for smoking cessation; counseling on smoking cessation maintenance and pharmacotherapy; management of abnormal results ② Exercise program: aerobic, muscle power ③ Smoking cessation education: theme-coping with craving, marketing of tobacco industry, introduction of national smoking cessation services, etc ④ Psychologic counseling: stress management, anger management
	Day 5	① Rounding and smoking cessation consultation: check smoking status (CO monitoring), craving for smoking, adjustment of smoking cessation medication; discharge medication, reservation of follow-up visit ② Psychologic counseling: formation of supporters for smoking cessation (family, friends) ③ Program evaluation, graduation: sharing about smoking cessation camp, oath for smoking cessation, endowment of graduation certificate

### 2.3. Covariates

Differences between the 2 age groups were determined by analyzing their demographic characteristics, smoking behaviors, and related characteristics as independent variables.

#### 2.3.1. Demographic characteristics.

Demographic characteris tics included gender, age, height, body weight, highest level of education, occupation, marital status, employment status, number of supporting people, underlying diseases such as hypertension, diabetes mellitus, and dyslipidemia. Age was classified into 2 categories (<65 years old and ≥65 years old) to determine the association between age and the success rate of smoking cessation. In order to evaluate education level, participants who had the highest educational attainment in elementary school, middle school, high school, university, and graduate school were scored 1, 2, 3, 4, 5, respectively. Employment status was classified into “employed” and “not employed”.

#### 2.3.2. Smoking behavior related characteristics.

Smoking behavior related characteristics included the age at which smoking commenced, the number of cigarettes smoked daily, total pack years, previous attempts to quit smoking, reasons for abstinence failure before and after intensive care smoking cessation camp, nicotine dependence, and carbon monoxide (CO) levels. The Fagerström test for nicotine dependence was used to assess independence for the participants.^[15]^

#### 2.3.3. Smoking cessation success rate.

The smoking cessation support center conducted a smoking cessation success evaluation at 4 weeks, 6 weeks, 12 weeks, and 24 weeks after completing the intensive care smoking cessation camp. Participants were monitored to maintain smoking cessation by self-reporting or by measuring their CO expiration level or by cotinine testing.

### 2.4. Statistical analysis

To evaluate the difference in success rates between the elderly and nonelderly age groups, a frequency analysis and chi-squared test were performed. Student t-test and chi-squared tests were performed to determine differences in underlying factors between elderly and nonelderly smokers. We considered *P*-values of <0.05 to be statistically significant. Analyses were performed using a computer-based statistical software package (Statistical Package for Social Sciences [SPSS] version 18.0; SPSS, Chicago, IL).

## 3. Results

Results at the 6-month follow-up showed that 56 of 107 (52.3%) elderly smokers and 109 of 244 (44.7%) nonelderly smokers continued to abstain from smoking. Elderly and nonelderly smokers showed comparable success rates at the 6-month follow-up.

Table 2 presents a comparison of demographic, smoking-related, and smoking cessation-related characteristics between the 2 age groups, comprising 94.3% of nonelderly and 98.1% of elderly subjects. The mean age was 54.22 years for the nonelderly and 69.58 years for the elderly. The general education level was higher in the nonelderly group. The mean education score was 3.57 for the nonelderly and 3.33 for the elderly. The proportion of married participants was 82% in the nonelderly age group and 90.7% in the elderly group. The proportion of employed participants was 65.2% in the nonelderly and 24.1% in the elderly, group. Nonelderly subjects tended to start smoking at an earlier age. They smoked more cigarettes per day, had higher nicotine dependence, and reported higher CO values than the elderly. The proportion of elderly patients with underlying diseases, such as hypertension, diabetes mellitus, and dyslipidemia, was higher than that of the nonelderly patients with underlying diseases.

**Table 2:** Comparison of demographic and smoking-related characteristics between 2 age groups.

Variable	Nonelderly (n = 244)	Elderly (n = 107)	*P* value
Percent of men	94.3% (230/244)	98.1% (105/107)	0.11
Age	54.22 ± 7.45	69.58 ± 4.33	<.001*
Height	169.95 ± 6.67	167.97 ± 4.84	0.006†
Body weight	73.18 ± 13.00	67.98 ± 8.06	<.001*
Education level	3.57 ± 0.78	3.33 ± 1.13	0.02‡
Marital status	82% (200/244)	90.7% (97/107)	<.001*
Employment status	65.2% (107/164)	24.1% (19/79)	<.001*
Number of supporting people	1.59 ± 1.04	1.60 ± 0.97	0.91
Exercise	64.0% (155/243)	71.8% (74/103)	0.30
Alcohol intake within 1 year	85.5% (200/241)	77.4% (82/106)	0.06
Cigarettes/day	23.83 ± 9.06	19.66 ± 9.45	<.001*
First smoking age	19.89 ± 3.60	21.82 ± 5.26	<.001*
Total pack years	38.16 ± 16.93	44.61 ± 22.31	<.001*
Having previous trial	43.9% (107/244)	51.4% (55/107)	0.47
Nicotine dependence	5.80 ± 2.23	4.93 ± 2.57	<.001*
Carbon monoxide value	9.73 ± 8.51	7.45 ± 6.02	0.01†
Hypertension	33/244 (13.5%)	37/107 (34.6%)	<.001*
Diabetes mellitus	35/244 (14.3%)	27/107 (25.2%)	0.01†
Dyslipidemia	25/244 (10.2%)	23/107 (21.5%)	<.001*

**Figure 2.**
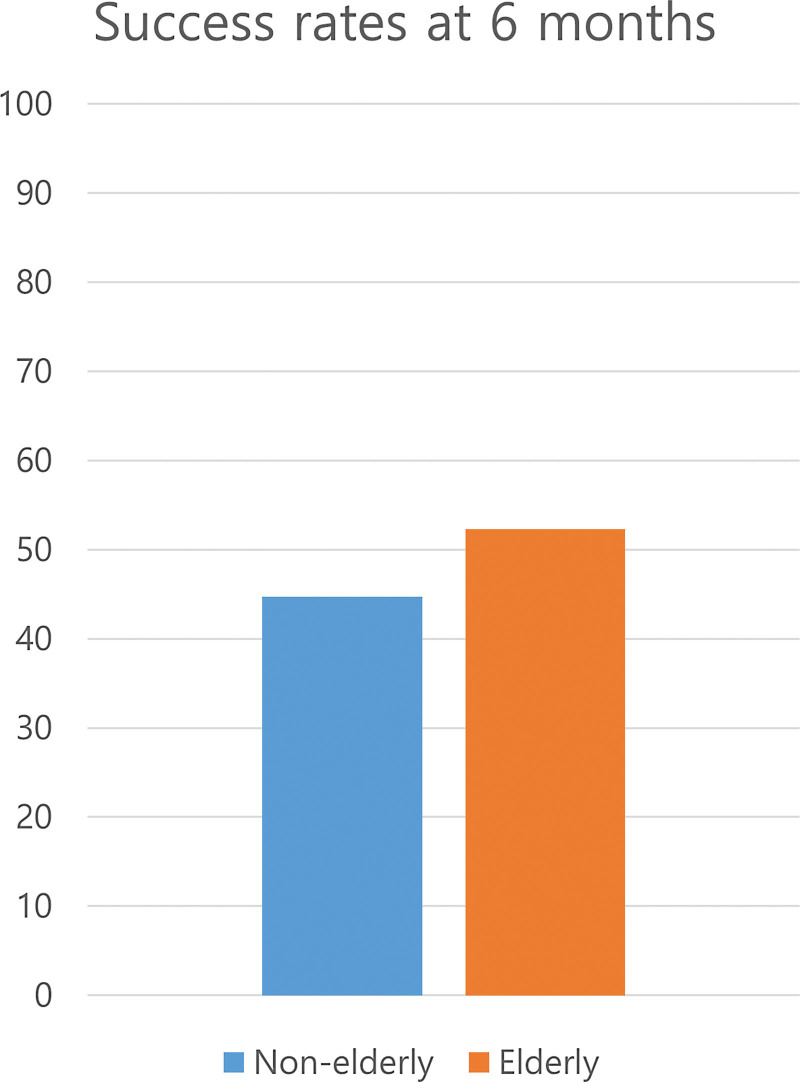
This figure graphically expresses the difference in smoking cessation success rates between the elderly and nonelderly 6 months after discharge from the smoking cessation camp. Among the elderly, 56 out of 107 smokers (52.3%) and 109 out of 244 smokers (44.7%) in the nonelderly group succeeded in quitting smoking. The difference between the 2 groups was statistically comparable.

Table 3 shows success rates of smoking cessation in the 2 age groups. A total of 269 out of 351 participants maintained smoking cessation at 4 weeks; this proportion decreased to 263 of 351 people at 6 weeks. Fewer participants were successful at later periods: 203 of 351 people continued to abstain from smoking at 12 weeks and 165 of 351 people did so at 24 weeks. As shown in Figure 2, the success rate of smoking cessation at 6 months was higher in the elderly than in the nonelderly. However, the difference was not statistically significant. (*P* value = 0.183)

**Table 3:** Success rates of smoking cessation in the 2 age groups.

Follow-up	Nonelderly	Elderly	Total	*P* value
4-week cessation	181/244 (74.2%)	88/107 (82.2%)	269/351	0.100
6-week cessation	178/244 (73.0%)	85/107 (79.4%)	263/351	0.197
12-week cessation	133/244 (54.5%)	70/107 (65.4%)	203/351	0.057
24-week cessation	109/244 (44.7%)	56/107 (52.3%)	165/351	0.185

Table 4 lists the factors underlying cessation failure in the 2 age groups before admission to the intensive care smoking cessation camp. The most common causes were stress and temptation, occurring in one-third of all smokers. The second most common cause was withdrawal symptoms. Table 5 presents factors contributing to cessation failure after the intensive care smoking cessation camp. Stress and temptation were the most common causes, followed by withdrawal symptoms.

**Table 4:** Factors contributing to cessation failure in the 2 age groups before admission to the intensive care smoking cessation camp.

Cause	Nonelderly	Elderly	*P* value
Stress and temptation	112/244 (45.9%)	34/107 (31.7%)	0.095
Withdrawal symptoms	45/244 (18.4%)	15/107 (14.0%)	0.311
Others	10/244 (4.1%)	5/107 (4.8%)	>0.99

**Table 5:** Causes of cessation failure in the 2 age groups after the intensive care smoking cessation camp.

Cause	Nonelderly	Elderly	*P* value
Stress and temptation	72/93 (77.4%)	22/30 (73.3%)	0.647
Withdrawal symptoms	19/93 (20.4%)	7/30 (23.3%)	0.735
Others	2/93 (2.2%)	1/30 (3.3%)	0.571

## 4. Discussion

This study investigated the differences in success rates of smoking cessation between elderly and nonelderly groups. Elderly individuals tended to maintain a smoking cessation rate comparable to that of nonelderly individuals. During the 6-month follow-up, 56 of 107 (52.3%) elderly smokers continued to abstain from smoking compared with 109 of 244 (44.7%) nonelderly smokers.

Few studies have investigated the factors associated with smoking cessation among elderly smokers in Korea. One study evaluating the smoking characteristics of elderly smokers reported a 66.8% smoking cessation rate. The findings from this study were confounded by the age at which the smokers profiled their quit. On average, elderly past smokers reported quitting in their 50s, while some past smokers stopped smoking in their 20s and 30s.^[16]^ In France, a retrospective study of smoking cessation was conducted in elderly (≥60 years old) and nonelderly (<60 years old) smokers who had attended a smoking clinic. The abstinence rate at 12 months was 44.2% in the elderly and 32.9% in the nonelderly. Age > 60 years was associated with a higher success rate.^[17]^ Similarly, another study reported that elderly smokers attending smoking clinics in Taiwan were as likely to achieve long-term abstinence as other adult smokers. Prolonged 36-month abstinence rates were 20.1% and 15.3% in the elderly and nonelderly participants, respectively.^[7]^

In the Republic of Korea, 65% of adult smokers intend to quit. Most try to quit without assistance from others. However, 84.7% of those who attempt smoking cessation fail because of a lack of willpower.^[18,19]^ Cessation failure is attributed to stress, temptation, and withdrawal symptoms. In our study, the most common causes of failure in both age groups were stress and temptation. This response was consistent with the findings of a previous study demonstrating that the use of willpower is an important factor in smoking cessation. This supports the benefit of participation in an intensive care smoking cessation camp, where smokers are motivated and empowered to quit smoking.^[14,20]^ Abstinence rates in intensive care smoking cessation camps are higher than those in smoking clinics. Thus, encouraging unmotivated smokers to participate in an intensive care smoking cessation camp could be an option for treatment.

Another notable finding of our study was that, compared to the nonelderly group, the elderly showed a higher rate of success in quitting smoking, despite their lower education levels. This is in contrast to previous studies showing that people with higher education levels were more likely to quit smoking.^[21,22]^ This may be because the education provided in the smoking cessation camps was effective, or it may be because the elderly pay more attention to their health as underlying diseases such as hypertension and diabetes mellitus are more common. The results of our study are expected to have an impact on policies to expand the target for smoking cessation camps in the future.

Our study had some limitations. First, this was a single center, retrospective study. Some important factors cannot be measured, which may act as confounders. Second, the sample size is relatively small which may have caused sampling bias and affected the validity of the findings. Third, since our study only targeted smokers admitted to intensive care smoking cessation camps, it is difficult to generalize the results to all smokers. Further multicenter studies are needed to identify the characteristics of smoking cessation among general smokers who are not admitted to an intensive care smoking cessation camp. Despite these limitations, our study showed that the elderly tend to quit smoking at rates comparable to those of the nonelderly; this finding was consistent with a previous study reporting that old age was related to successful smoking cessation.^[7,23]^ Since there are few data on smoking cessation camps for the elderly, it is possible to provide an objective perspective based on the results of the national smoking cessation camp follow-up.

In conclusion, smoking cessation is recommended for the elderly because of the remarkable success rates irrespective of age. Admission to intensive care smoking cessation camps is beneficial for smokers intending to quit smoking including the elderly. Thus, we should strongly recommend that elderly smokers, as we do for the nonelderly, stop smoking.

## Author contributions

Conceptualization: Jae-Kyeong Lee, Yu-Il Kim

Data curation: All of authors

Formal analysis: Jae-Kyeong Lee, Yu-Il Kim

Investigation: Sun-Seog Kweon, Yong-Soo Kwon, Yu-Il Kim

Methodology: Jae-Kyeong Lee, Yu-Il Kim

Project administration: Jae-Kyeong Lee, Yu-Il Kim

Supervision: Sun-Seog Kweon, Yong-Soo Kwon, Yu-Il Kim

Validation: Sun-Seog Kweon, Yong-Soo Kwon, Yu-Il Kim

Writing—original draft: Jae-Kyeong Lee, Yu-Il Kim

Writing—review & editing: All authors

Approval of final manuscript: All authors

## Acknowledgment

The authors thank the intensive care smoking cessation camp at Chonnam National University Hospital.

## Footnotes

* *P* <.001.

† *P* <.01.

‡ *P* < .05.
